# Acute isolated traumatic anterior pisiform dislocation in an adult: A case report

**DOI:** 10.1016/j.tcr.2026.101317

**Published:** 2026-03-03

**Authors:** Devansh Shukla, Jannik Engelhardt, Philipp Eckardt, Philipp Kobbe, Stefan Delank, Matthias Aurich

**Affiliations:** a Universitätsklinik Halle (Saale), Halle (Saale), Germany; b BG Klinikum Halle, Halle (Saale), Germany; c Klinikum Altenburger Land, Altenburg, Germany

**Keywords:** Emergency medicine, Pisiform, Trauma management, Upperlimb, Flexor carpi ulnaris, Radiographs

## Abstract

Isolated pisiform dislocation without associated carpal bone injuries is a rare clinical entity, with few reported cases. This report describes a 23-year-old, right-handed male who presented in March 2024 with acute anterior pisiform dislocation following indirect trauma while lifting furniture. The patient experienced pain, mild swelling, and tenderness in the distal ulnar region of the right hand, with initial radiographs confirming the dislocation. Spontaneous repositioning occurred during transfer to a trauma hospital after cast immobilization, eliminating the need for further imaging or invasive treatment. The patient had no history of hypermobility syndrome. Early physiotherapy facilitated full recovery of range of motion. After one-year follow-up, the patient showed a Quick-DASH score of 0, indicating complete functional recovery; therefore, no control radiographs were performed to avoid unnecessary radiation exposure in this young patient. The Quick-DASH (Disabilities of the Arm, Shoulder and Hand) score of 0/100 confirms excellent objective and subjective outcome with no residual disability. The injury likely resulted from wrist hyperextension and forceful flexor carpi ulnaris contraction. Diagnosis typically relies on clinical findings and radiographs, though advanced imaging may be required in ambiguous cases. Treatment options include immobilization, closed reduction, open reduction with internal fixation, or pisiform excision, depending on the case. This report underscores the importance of considering isolated pisiform dislocation in young patients with ulnar-sided wrist pain post-trauma and highlights the efficacy of conservative management when spontaneous reduction occurs.

## Introduction

**Fig. 1:**
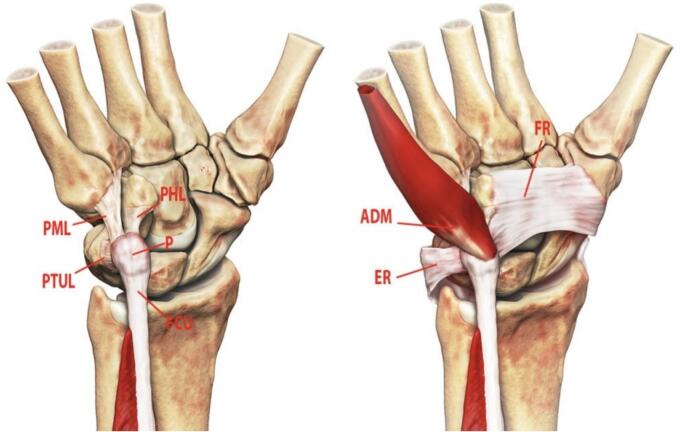
The primary stabilizers of the pisiform (P) are the flexor carpi ulnaris (FCU) tendon, pisohamate ligament (PHL), pisometacarpal ligament (PML), and pisotriquetral ulnar ligament (PTUL). Secondary stabilizers include the flexor retinaculum (FR) and extensor retinaculum (ER). The abductor digiti minimi (ADM) arises primarily from the pisiform.

Isolated pisiform dislocation without concomitant carpal bone injuries is an exceedingly rare condition, with only a few documented cases in the literature [1], [2], [3], [4]. The pisiform, a sesamoid bone within the proximal row of the carpus, articulates with the triquetrum and hamate. Its articular surface is horizontal, and its stability relies primarily on the flexor carpi ulnaris (FCU) tendon, ulnar pisotriquetral ligament, pisometacarpal ligament, and pisohamate ligament [5]. Secondary stabilizers include the flexor retinaculum (FR) and extensor retinaculum (ER), while the abductor digiti minimi (ADM) originates from the pisiform (Fig. 1).

(Image source: [5]).

The FCU tendon, attached to the anterior aspect of the pisiform, pulls the bone proximally during contraction, aiding wrist flexion with support from the pisohamate and pisometacarpal ligaments. Two mechanisms of acute pisiform dislocation have been proposed: (1) direct force applied to the pisiform, or (2) indirect force from wrist hyperextension combined with strong FCU contraction, displacing the pisiform proximally [2], [3]. This report presents a case of acute isolated traumatic anterior pisiform dislocation in a young adult, highlighting its diagnosis and management.

## Case report

**Fig. 2:**
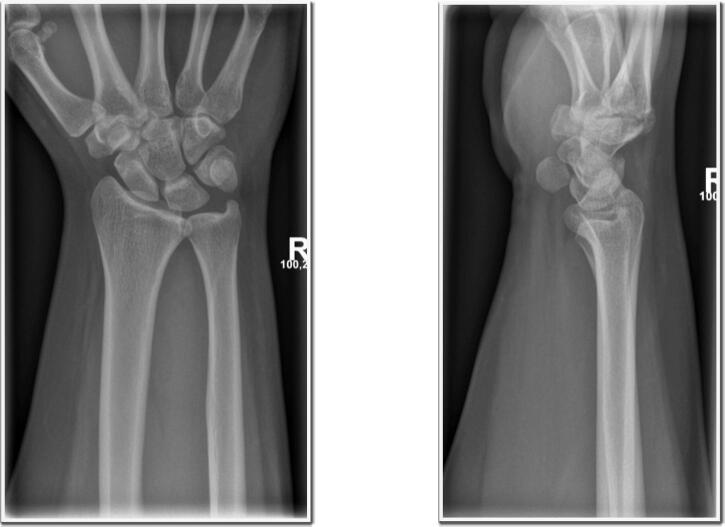
Radiographic evidence of anterior pisiform dislocation in the right hand (Universitätsmedizin Halle, Saale).

In March 2024, a 23-year-old, right-handed male presented to the emergency department with pain, mild swelling, and tenderness in the distal ulnar region on the ventral side of his right hand. The patient reported lifting furniture (approximately 10 kg) at work when he heard a popping sound, suggesting an indirect traumatic mechanism. He had no history of hypermobility syndrome or previous wrist complaints. He sought medical attention within 2 h of the injury. Clinical examination revealed no neurological deficits or additional symptoms. Initial radiographs confirmed an anterior pisiform dislocation (Fig. 2).

**Fig. 3:**
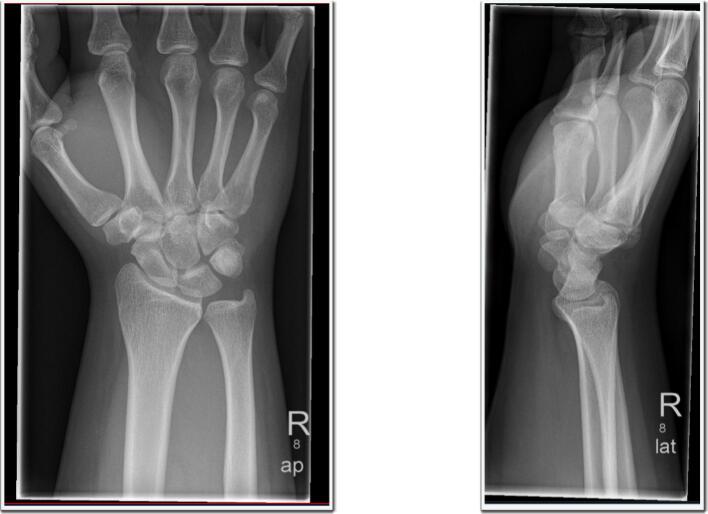
Radiographic evidence of spontaneous pisiform repositioning in two planes (BG-Klinik, Halle, Saale).

The patient was managed with cast immobilization and transferred to a specialized trauma hospital. Repeat radiographs upon arrival showed spontaneous repositioning of the pisiform, with no evidence of persistent dislocation (Fig. 3). Given the lack of neurological symptoms and spontaneous reduction, further imaging (e.g., CT or MRI) was deemed unnecessary. Early physiotherapy was initiated, and follow-up assessments demonstrated full restoration of range of motion without restrictions. After the one-year follow-up, the patient reported no pain or functional limitations and showed a Quick-DASH score of 0. The Quick-DASH score of 0 points after 12 months post-injury documents complete return to pre-injury functional level and absence of any disability. Consequently, no follow-up radiographs were obtained to minimize radiation exposure in this young individual.

## Discussion

In this case, the pisiform dislocation resulted from indirect trauma during furniture lifting, likely due to sudden wrist hyperextension and forceful FCU contraction [2], [3]. Clinically, pisiform dislocation typically presents with pain, swelling, and tenderness over the ulnar wrist, as observed in our patient. Diagnosis relies on the injury mechanism and physical findings, though standard anteroposterior (AP) and lateral radiographs may not always detect the dislocation [1], [4]. Oblique views or comparison with the contralateral wrist can enhance diagnostic accuracy, while CT and MRI provide definitive assessment of bone and soft tissue injuries, respectively [3], [5]. In this instance, the emergency trauma surgeon confirmed the diagnosis using conventional AP and lateral X-rays, which clearly demonstrated the dislocation.

Treatment options for pisiform dislocation include closed manipulative reduction with immobilization, open reduction with internal fixation (ORIF), or pisiform excision [1], [2], [3]. Immobilization following closed reduction remains debated, with some advocating for forearm pronation to relax the FCU and stabilize the pisiform [4]. In our case, the wrist was immobilized in a neutral position during transfer, yet spontaneous reduction occurred, negating the need for further intervention. Immobilization followed for 2 weeks followed by early functional physiotherapy. ORIF is reserved for failed closed reductions or delayed diagnoses, which can lead to complications such as recurrent dislocations, chronic pain, or pisohamate/pisotriquetral arthritis [2], [5].

Wrist injuries are common in emergency settings, yet isolated pisiform dislocation is rare and easily overlooked. In young patients with ulnar-sided wrist pain following trauma, this condition should be considered in the differential diagnosis. Prompt referral to an orthopedic specialist is critical for early diagnosis and optimal management.

## CRediT authorship contribution statement

**Devansh Shukla:** Writing – review & editing, Writing – original draft, Methodology, Formal analysis, Data curation, Conceptualization. **Jannik Engelhardt:** Data curation. **Philipp Eckardt:** Formal analysis, Data curation, Conceptualization. **Philipp Kobbe:** Writing – review & editing, Validation, Supervision. **Stefan Delank:** Writing – review & editing, Validation, Supervision. **Matthias Aurich:** Writing – review & editing, Validation, Supervision, Methodology, Formal analysis.

## Funding

The author(s) received no financial support for the research, authorship, and/or publication of this article.

## Declaration of competing interest

The author(s) declared no potential conflicts of interest with respect to the research, authorship, and/or publication of this article. Disclosure forms for all authors are available online.
